# Efficacy of quercetin-like compounds from the mistletoe plant of *Dendrophthoe pentandra* L. Miq, as oral random blood sugar lowering treatment in diabetic rats

**DOI:** 10.1080/01652176.2024.2372090

**Published:** 2024-06-29

**Authors:** Mochamad Lazuardi, Qonita Kurnia Anjani, Aniek Setya Budiatin, Tjuk Imam Restiadi

**Affiliations:** a Subdivision the Veterinary-Pharmacy Science, Faculty of Veterinary Medicine, Universitas Airlangga, Surabaya, Indonesia; b School of Pharmacy Queen’s University, Belfast, Northern Ireland; c Material Division, Faculty of Pharmacy, Universitas Airlangga, Surabaya, Indonesia; d Faculty of Veterinary Medicine, Universitas Airlangga, Surabaya, Indonesia

**Keywords:** *Benalu duku*, Genistein, Health-lifestyle, Insulin, Mistletoe, Morin, Quercetagin, Sugar blood

## Abstract

**Background:** Mistletoe is an herb that grows on duku plants (Lancium demosticum) and is known as benalu duku (BD) in Indonesia. It is predicted to have benefits such as anticancer or antiviral properties, and it is also thought to have anti-diabetic pharmacological activity. Quercetin-like compounds (QLCs) are secondary metabolites with antidiabetic activity that are expected to lower blood sugar levels in animals after oral administration.

**Objective:** This study aimed to analyze the ability of QLCs to reduce random blood sugar levels using experimental animals as clinical models.

**Material and methods:** The research method used was exploratory, which used a before–after test model, and observations were made on the random blood sugar levels after treatment. Secondary metabolites were extracted from BD leaves, which were then screened. Diabetes was induced in 30 rats (Rattus norvegicus) by the administration of streptozotocin at 0.045 mg/g body weight daily for 2 days. The antidiabetic effects of the secondary metabolite at doses of 0.5 mg/kg body weight (twice a day) when administered orally for up to 5 days were tested in diabetic rats. The random sugar levels (mg/dL) were measured using a One Touch Ultra Plus medical device for observation of randomized blood sugar levels. Results and novelty: The results revealed that the secondary metabolite, as an analyte from the BD leaf extract, can significantly reduce random blood sugar levels.

**Conclusion:** The secondary metabolite extracted from BD, could be used to treat diabetes in rats.

## Introduction

The use of natural compounds for medicinal purposes in a healthy lifestyle is included in the Sustainable Development Goals. One of them is the use of components such as alkaloids, tannins, plant enzymes, and other useful compounds. These components are widely found in plants, especially within the medicinal plant category (Heinrich et al. 2021). Among the most interesting medicinal plant types is a parasitic plant known as mistletoe. Mistletoe is characterized by its hard stems and leaves that are not jagged, and the plant flowers and thrives after the rainy season (Silveira and Boylan 2023). This generally grows and develops on its host plant and generally has various pharmacodynamic properties, one of which is *Dendrophthoe pentandra* (L.) Miq, which grows on the host plant *Lancium domesticum*.

Recent research has demonstrated that the percolation of mistletoe leaves from *Dendrophthoe pentandra* (L.) Miq, or *benalu duku* as it is known in Indonesia, is known to potentially have anticancer, antivirus, and antidiabetic activity (Lazuardi et al. 2022; Kong et al. 2023). Its anticancer properties have been previously reported, especially against myeloma cells, while its antivirus activity has been reported to have antiviral activity against the Newcastle Diseases virus (Lazuardi et al. 2023). In addition, it is known that heat maceration of the stems of these plants can be used as a mosquito repellent, where the appropriate application is in the form of a topical jelly. A recent report has shown that the DB stem contains essential compounds that have repellent power against *Aedes aegypti* mosquitoes. These essential compounds are suitable for human skin and do not irritate the epidermis (Awang et al. 2023; Rachmawati 2023). Thus, the active compounds could potentially be used for cosmetic preparations in humans.

It has not been reported as an antidiabetic in either experimental animals or humans using specific percolation stratified maceration techniques, for example, solvent methanol, ethyl acetate, and normal hexane. The multilevel maceration technique produces compounds rich in polyphenols and a small amount of condensed and hydrolyzable tannins (Lazuardi et al. 2022). This is possible considering that the plant is known to contain quercetin-like compounds (QLCs) as secondary metabolites or analytes that have a protective role on the surface of eukaryote cells. The elements of analytes that are important for life in eukaryotic cells are (a) polyphenol; (b) donor electron groups, such as carbon ions and nitrogen ions; (c) positrons as electron-attracting balancing groups, such as hydrogen ions; and (d) other groups that balance the forces of electrostatic attraction between ions but are categorized as electron binding groups. It is known that electrostatic bond balancing elements between ions can directly maintain the balance of breaking bonds because of addition or substitution reactions from donor and electron acceptor ions that have very strong electrostatic forces. Among these groups are heavy metal ions. If there is a reaction with heavy metal ions, then eukaryote cells cannot survive naturally, which will give rise to uncontrolled cell growth and the onset of degenerative disease (Alam et al. 2022; Rachmawati 2023).

Tannins have multifunctional roles in plants, including (a) astringency, (b) antioxidants, (c) cofactors in glycolysis, and (d) inhibition of kinetoplast activity in the Trypanosoma lifecycle. As an antioxidant, O^2−^ are ions of the polyphenolic compounds of QLCs and are known to bind with the ions of H^+^ and C³^+^ as a part of the lipid bilayer of cells to protect the cell pores from other external destruction compounds (Tong et al. 2021). This phenomenon is a protective mechanism of cells from external chemical substances with negatively charged ions with a greater energy potential than ions O (Zhang et al. 2021). Although analytes serve as protectors of eukaryotic cells, they also affect chemical components that can damage the cells (Lazuardi et al. 2022; Michala and Pritsa 2022). The relationship between the role of polyphenol and tannin elements causes the superiority of the secondary metabolites from plants that are used as *Cardinale remediums*.

The target eukaryotic cells are pancreatic β-cells located in the islets of Langerhans. These cells are highly susceptible to chemical compounds that result from drug metabolism *via* the analytes. Drug metabolites can bind to water-soluble molecules, but pancreatic β-cells can be protected, and insulin secretion may continue because the protective power of analytes can balance the acidic base of the internal cytoplasmic solution. If pancreatic β-cells grow excessively, this will stimulate growth between cells and produce a sufficient amount of insulin. Under these conditions, high levels of blood sugar in the blood will be rapidly degraded (Hogrebe et al. 2021; Lim et al. 2022). These advantages allow for the use of active drug compounds that are rapidly metabolized by the body, and the resultant metabolites do not cause drug residues.

Based on the description above, this study considers the effect of analytes on random sugar levels in experimental diabetic animals. Furthermore, this study assessed the results of the administration of analytes with targeted antidiabetic action *via* posttherapy monitoring of the random blood sugar levels. The measurements are performed based on the bond, which will be determined from the absorbance; thus, if the bond is weak, a reduced value will be exhibited; however, if the bond is too strong, the measurement cannot be accurately determined. Therefore, we did not investigate the value of glycosylated hemoglobin using the test kit because it had this weakness (Aba and Asuzu 2018).

## Material and methods

### Research design

**Figure 1.**
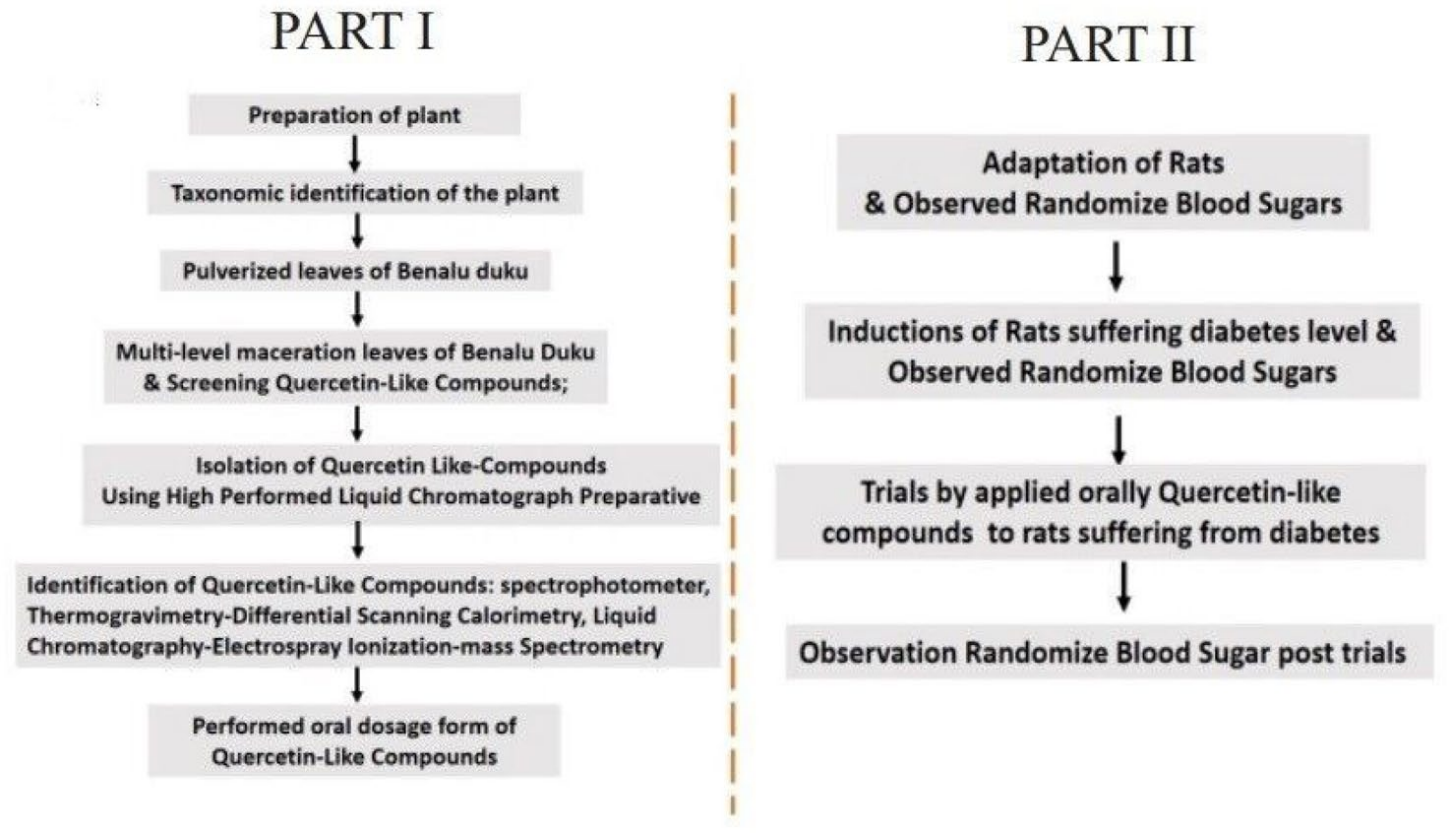
Research flowchart for the study, ‘efficacy of quercetin-like compounds from the mistletoe plant of *Dendrophthoe pentandra* (L). Miq, as oral random blood sugar lowering treatment in diabetic rats’.

This study was performed in two stages: the initial stage isolated the analyte compounds from the benalu duku (BD) leaf extracts, and the stage that followed was performed on rats as experimental animals using a before–after trials study design. The research flowchart is presented in Figure 1.

### Research schedule and location

The research procedures began in January 2022, were intensively performed since December 2022, and ended in the third week of October 2023. All research was conducted at the Research Center for the Application of Veterinary Pharmacy Sciences, Faculty of Veterinary Medicine, Universitas Airlangga. Simultaneously, the Indonesian Veterinary Pharmacy and Pharmacology Association was established under the control of the Indonesian Veterinary Association.

### Plants and animal ethics approval

Mistletoe plants of BD, growing on *Lancium domesticum* as its host plant, were obtained from Muara Enim Regency in the regions of South Sumatra Province. Geographically, it is located between 4° and 6° south latitude and 104° and 106° east longitude. The leaf samples were collected from BD plants from January 2022 to March 2023, considering that mistletoe growth was more developed than in the summer at the end of the rainy season. BD was taxonomically verified by the National Biological Institute, National Innovation Research Agency, Republic of Indonesia. A plant validity certificate was received on 7 June 2022 (No. B-1679/II.6.2/D1.05.07/6/2022).

*Rattus norvegicus* rats were obtained from a commercial experimental animal company in Surabaya-Indonesia, and animal ethics approval was provided by the Faculty of Veterinary Medicine at Airlangga Universitas on 14 April 2023 (No. No: 1.KEH.060.04.2023).

### Sample size and streptozocin for inducing diabetes in rats

The number (N) of rats used in this study was calculated using the following formula; where the standard deviation (S) and the error of tolerance (E) were set at 0.28 and 0.1, respectively. The value $Z_{1}-\frac{\alpha }{2}$ were determined to be 1.96 at the significant level of Z_0.95_ (Pourhoseingholi et al. 2013; Andrade 2020; Althubaiti 2023).

(1) $$N=\frac{{\left(Z_{1}-\frac{\alpha }{2}\right)}^{2}\times {\left(S\right)}^{2}}{E^{2}}$$

In this study, the number of rats was determined to be *N* = 30.118, which was rounded down to 30. Thus, the number of rats required was 30, and they were healthy males aged six months with an average body weight of 169 G. Streptozotocin of analysis grade was purchased from Sigma-Aldrich Corp. (catalog number S0130), which was dissolved in an aqua injection solution under aseptic conditions to a concentration of 100 mg/1000 μL and placed in a sterile injection vial with dark glass as the trial preparation. The dose was determined to be 0.045 mg/g body weight injected intraperitoneally every day for two days.

### Procedure for compound extraction from plants

Leaves of BD plants (6 kg) were collected, washed with running water, and air-dried in a cool place without exposure to direct sunlight for 24 h. The leaves were then crushed using a grinder and sieved using a sieve size 000. Then 900 g of BD leaf powder was macerated using the moving method for five days with an analysis-grade methanol solvent (solid–liquid) from EMPARTA^®^ ACS Supelco Corp., Darmstadt, Germany (catalog number 107018) to obtain the macerate-containing flavonoids. Further maceration was performed to remove other polyphenolic interfering compounds using a 50:50 mixture ratio of analysis-grade water-n-hexane (liquid–liquid) from EMPARTA^®^ ACS Supelco Corp. (catalog number 107023) solvent for two days. Separation was continued using a separator flask, which was expected to contain flavonoid components that still contained tannins. The maceration products were dried using a vacuum evaporator with a temperature setting of 37–40 °C.

The macerated product, after separation and drying, was tested for its flavonoid content. The Willstatter test was used in a solution of 1 mL of macerate and added to 2–4 mL of concentrated analysis-grade HCL (catalog number 30721-2.5 L, Sigma-Aldrich Corp.), which was then shaken vigorously. Approximately 0.25 mg of Mg was added (catalog number S7562917814, Merck Corp.), and it was further shaken. The results showed a change in color from the original color of the forage macerate to red or orange. The results of the flavonoid tests were confirmed. The Bate-Smith test was performed by adding 250 mg of macerates with 2–5 drops of concentrated H_2_SO_4_ and placing it in a water bath at 100 °C for 20 min until the color changed from the original color green to orange. The next stage tested the tannin content by adding 2–3 drops of 1 iron (III) chloride hexahydrate (catalog number 1.03943.0250, Merck Corp.) to 1 mL of the macerate in water injection from Ika Pharmindo Putra Mas Jakarta-Indonesia (w/v) and vigorously shaken. As a control, a tannin standard (identity number: tannic acid, Nitra Kimia, Malang-Indonesia) was used to compare the sample tests. The procedure of the control for the determination of tannic acid was similar to that of tannic acid from the samples. The result obtained was a color change reaction from the original green to a red-dark color as a result of the appearance of CHCL_3_.

### Screening contents of quercetin-like compounds

Screening for QLC content was performed using thin-layer chromatography (TLC) with TLC silica-gel 60 F_254_ catalog number HX72274254 from Merck Corp. and was conducted using quercetin standards to compare the dried macerate. The mobile phase solution was determined in the form of serial blinding of chromatograph methanol (LiChrosolv catalog number 1.06018.2500, Supelco Corp.) and water (LiChrosolv catalog number 1.15333.2500, Supelco Corp.,) with percentage fractions of 50:50, 60:40, 70:30, 80:20, 90:10, and 100:0, respectively (Lazuardi et al. 2023).

The initial step in performing TLC analysis was to saturate the elution chamber by inserting a filter paper submerged in the mobile phase solution and closing the chamber. This continued for up to 1 h until the entire filter paper appeared wet. The next step was to prepare the TLC paper to mark the a of the analyte drops and standard and to set the elution distance. The analyte dropper device uses heparin-free microhematocrit capillary glass pipes. The analytes were then spotted on TLC paper, and the standard quercetin-spotting method was used. The elution was performed using a TLC chamber and was halted according to the elution limit. The TLC paper was dried and sprayed using analysis-grade 5% AlCl_3_ (catalog number 231-208-1 Sigma-Aldrich Corp.) dissolved in aqua injection (w/v) and monitored using a UV-viewer at wavelengths of 254 and 365 nm (type UV-OC-02, GriyaLab Corp., Jakarta Timur-Indonesia). Next, the refractive index (R_f_) was calculated between the analyte and standard QLCs.

### Isolation-identification of analytes and preparation for oral application

Isolation was performed using a preparative High-Performance Liquid Chromatography (HPLC) device with an Agilent 1260 Infinity Diode Array Detector (DAAD) binary pump system. The columns used were an Infinity Lab ZORBAX SB-C_18_ 50 × 10.0 mm 5 µm (part-number Agilent column 446905-802). The mobile phase was water for chromatography and methanol at a ratio of 40:60 at a flow rate of 2 mL/min and a wavelength of 250 nm, and the mobile phase used was water and methanol for chromatography at a ratio of 40:60 at a flow rate of 2 mL/min and a wavelength of 250, 370, 375.4, and (reference) 360 nm, with a stop time of 30 min. The autosampler collector was used at a time of 21–23 min after the injection of 50 μL of the analytes into the column. The standard used was quercetin from Sigma Corp. (catalog number: Q4951-10G). The QLCs were dried using nitrogen gas in a water bath at 40 °C.

For the identification of the QLCs, the results were compared with the quercetin standard using a Fourier-transform infrared spectrophotometer (FT-IR), thermogravimetry-differential scanning calorimetry (TG-DSC), and liquid chromatography-electrospray ionization-mass spectrometry (LC-ESI-MS). FT-IR was performed using a Perkin Elmer Spectrum One (PN 09934358 by the Kbr-Transmisi) with a parameter range of 4000–400 cm^−1^, a duration scan of 20 s, and a resolution of 4 cm^−1^. TG-DSC was performed using a Metler Toledo TG-DSC at a temperature of up to 1200 °C. The gas flow was regulated, and ambient air could be used under nitrogen or oxygen conditions. TG-DSC utilized a solid sample with a minimum of 1 g and a maximum dimension of 2 mm^2^. The scanning of TG-DSC was set at an interval specified by the run heat of 10 °C/s, and the last scan was performed at 150 °C.

The LC-ESI-MS used ultraperformance liquid chromatography-mass spectrometry (UPLC-MS) with the UNIFI software program (Waters Corp., MA, USA). The column was set on octadecyl-xylene C_18_ stainless steel (Agilent catalog number 5982-1111). The temperature of the column oven and autosampler injectors was set at 20 °C, and the injector volume capacity was set to 10 μL. The running parameters were adjusted along a gradient system using the mobile phase A (chromatograph acetonitrile catalog number 1.00030.4000 from Merck Corp.) containing 0.1% formic acid (catalog number 801081, Merck Corp.) and mobile phase B (chromatograph water containing 0.1% formic acid p.a.). The flow rate was adjusted to 0.6 mL/min. The MS parameters were adjusted to the time-of-flight (TOF) MS E mode using electrospray ionization (ESI) at +/− and an acquisition range of 50–1200 Da. The following analysis criteria were adopted: mass error reading analyte ≤5 ppm, isotope match m/z root mean square (RMS) ≤6 ppm, isotope match m/z RMS% ≤10%, and an analyte intensity of ≥300. For one fraction, the brake value was <4 for the fragment elucidation system.

For the oral application, dimethyl sulfoxide from Supelco Corp. (catalog number K53320252.129) was added to the analytes at a ratio of 1:2 to make a solution, which was then adjusted to the physiological pH range of 6.70–7.00 by the addition of NaOH 1% (w/v) or HCL 0.01 N. A pH meter was used to measure the acid–base balance of the oral analytes using a PHS/3D pH meter model PHS/3D/02 assembly (Shenzhen, China). A buffer solution at pH 7.00, pH 4.00, and pH 10.00 as well as a potassium chloride solution were used in a Certipur (catalog number: HC73815639, HC74125835, HC73963838, HC72700317, Merck Corp.). Oral analytes were filtered using Whatman Unifl syringe filters (catalog number 9910-1302, Cytiva, USA), at a diameter of 0.2 μm.

### Animal experiments

The room’s conditions and equipment were set up according to the requirements for the random blood sugar testing devices. The random blood glucose measuring device used was the Self-Monitoring of Blood Glucose OneTouch Ultra Plus Flex with testing strips (lot number 5605067, OneTouch Corp., USA). The devices have a minimum and maximum measurement range of glucose blood of between 70 and 600 mg/dL at a room temperature of 20 °C −25 °C and a relative humidity of 10–20%. The altitude at the testing facility was 10 ft (*r* = 3.048 m). An automatic reading was performed 10 s after a drop of blood was applied to the strip (Littmann et al. 2013; Zafra-Tanaka et al. 2022). The rats were handled as experimental animals and were adapted to the procedure of random blood sugar checks. The rats were housed under standard experimental conditions with *ad libitum* access to feed and water for one week for acclimatization. The feed was obtained from type 521 production PT (Charun Pokphand, Indonesia) and water from Le-Mineral (Indonesia). Random blood sugar (mg/dL) level tests were performed every morning and evening after feeding for three days *via* the coccygeal vein. Diabetes was then induced in the rats. Streptozotocin was injected daily in the afternoon for two days at a dose of 0.045 mg/g body weight. Thereafter, the drinking water of the rats was replaced with *syrupus simplex*, which contains 64–66% sugar, and additional dry food in the form of brown sugar were provided in the rat cages. After streptozotocin injection, random blood tests (mg/dL) were performed every afternoon for two days. Next, an oral solution of the analytes was administered using an oral sonde every 12 h for five days at a dose of 0.05 mg/g body weight. The specifications of the oral sonde devices are that the sonde body part has a length of 13 cm with a small bulb at the port of the oral sonde, which was obtained from https://shp.ee/xtfbrc9. In the final stage, all liquids were replaced with mineral water, and the sugar-containing solid feed was removed. The rats had difficulty exercising for 1 h each day for 14 days at 22 °C to 25 °C. Random blood glucose tests (mg/dL) were evaluated during the 14-day random blood test period.

### Statistical analysis

The analysis of the data included random blood sugar tests before the administration of analytes and the means thereof after the administration of analytes. A paired *t*-test analysis was performed for the data from before and after the administration of the analytes using the IBM Statistical Package for the Social Sciences for Windows, version 24 (IBM Corp., Armonk, N.Y., USA) with α = 0.95. The results of the data analysis of the random blood glucose values before and after the administration of the analytes are the answers to the research questions detailed in the Introduction section.

## Results

**Figure 2.**
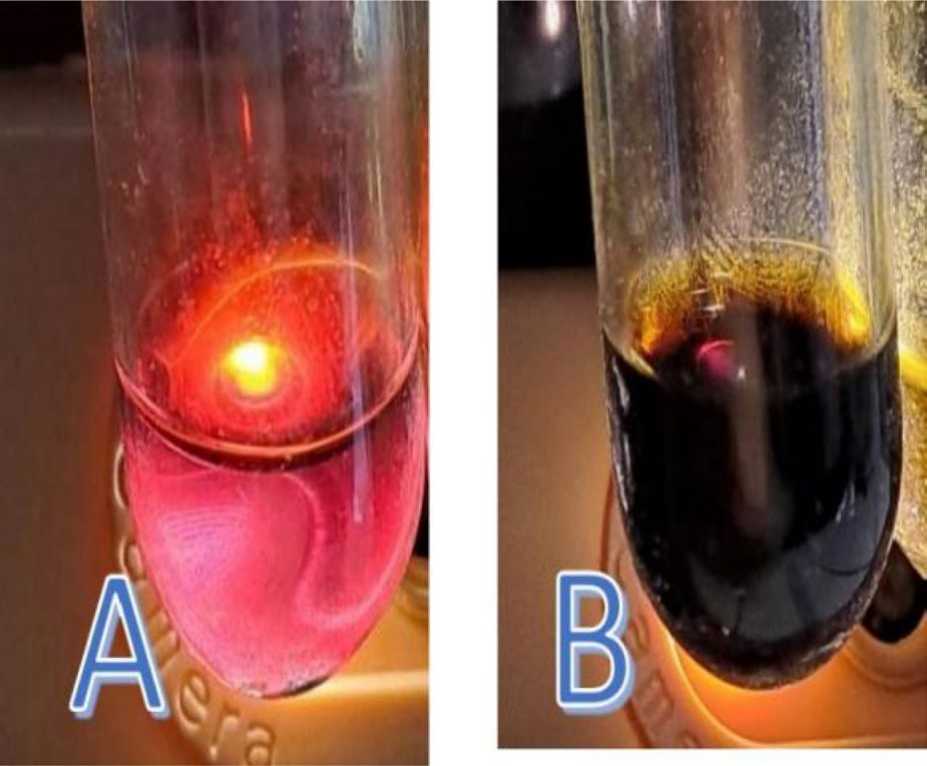
Test images showing the presence of flavonoids and tannins. (A) The presence of flavonoids was determined using the Bate-Smith method, which produced an orange color change. (B) The tannin test was determined using the qualitative method of adding concentrated sulfuric acid, which produced a red-dark color change.

The extracted macerate contained flavonoids and tannins, and quercetin was also found in the final macerate. The results of the Bate-Smith screening test showed that the final macerate contained flavonoids, as can be seen in the color change from the original greenish to orange. The test for the presence of tannins showed a color change from the original greenish color to a red-dark color (Figure 2).

**Figure 3.**
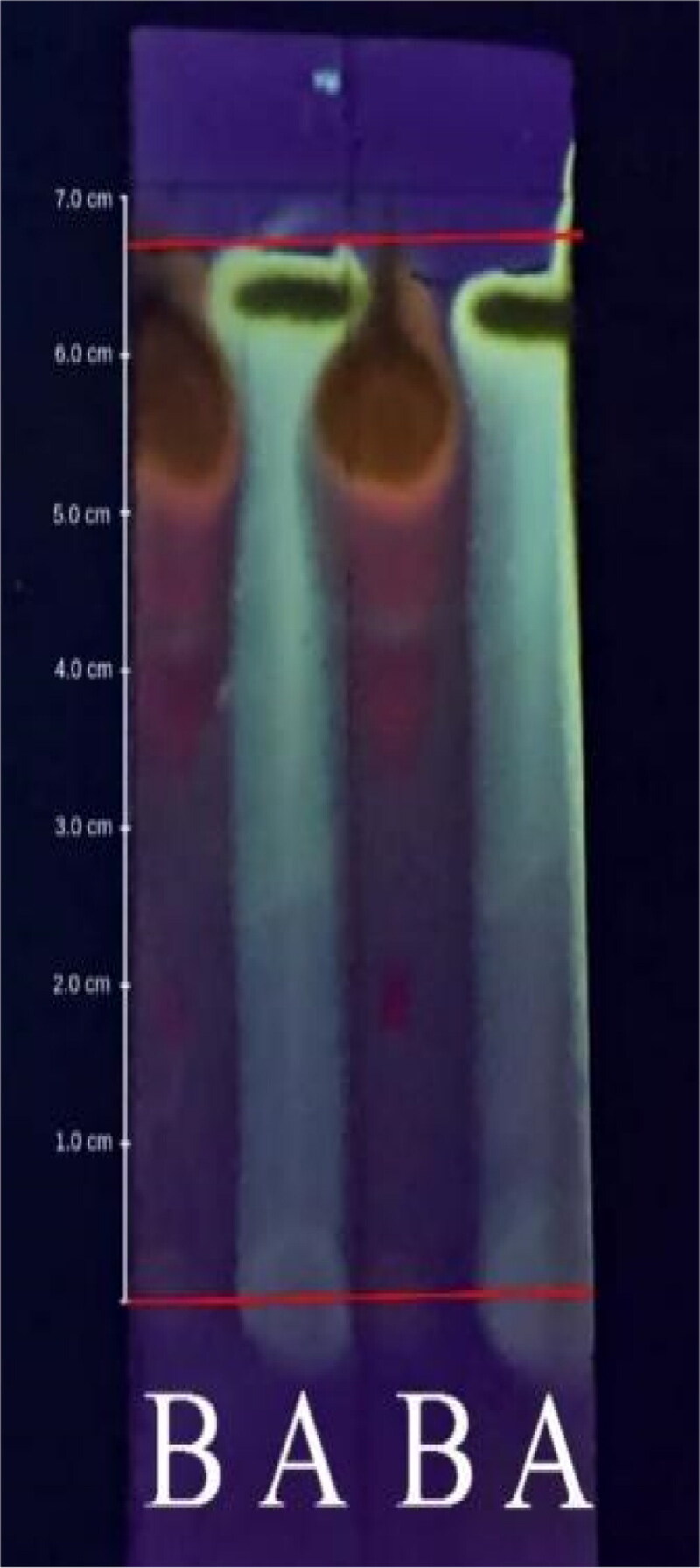
The elution on thin-layer chromatography was performed in duplicate and observed at 254 nm. Notation a is quercetin, and notation B is quercetin-like compounds in the mobile phase with methanol: water (40:60) at pH 6.97.

Screening results of the adsorption-partition properties between the flavonoid macerate and the quercetin standard using TLC with a mobile phase of methanol: water (60:40) exhibited an R_f_ value of 1. In Figure 3, the left represents the analyte, and the right represents the quercetin standard. During elution, there was no spot, and the boundary with the standard solution appeared to have the same landing time. Therefore, R_f_ = 1. The B notation represents the flavonoid-containing analytes, and the A notation represents the quercetin standard under saturated elution conditions, which was performed in duplicate at a wavelength of 254 nm. The experiment was performed twice, and the same Rf value was obtained under saturated conditions, which affected the adsorption-partition power of the stationary phase to produce an R_f_ value equivalent to the quercetin standard.

**Figure 4.**
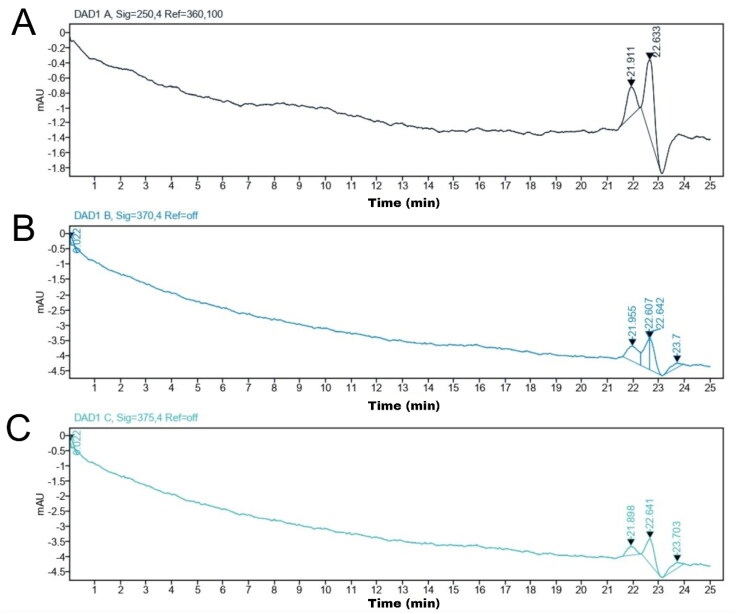
(A) Chromatogram of quercetin at 10 μg/mL in methanol at 250 nm; (B) chromatogram of quercetin-like compounds dissolved in methanol chromatography at 370 nm; and (C) 374.4 nm.

The results of the analysis of the collector fraction determination using an HPLC preparative device revealed that the quercetin standard had peak areas at retention times of 21.911 and 22.633 min and that the analytes had peak areas at retention times of 21.955, 22.607, and 22.642 min. Thus, the collector fraction includes the analytes at the retention time of the quercetin standard. The selected wavelengths also result in different peak areas. Figure 4 of the Preparative HPLC chromatogram shows that chromatogram A, as the quercetin standard at 10 µg/mL, exhibited a wavelength of 250 nm, while chromatograms B and C are analytes that were observed at wavelengths of 370 and 375.4 nm, respectively.

The isolation results revealed that the analyte compounds collected at retention time had color characteristics similar to those of the quercetin standard, namely yellowish. Thus, it can be determined that the compounds collected from the HPLC preparations have physicochemical properties similar to those of the quercetin standard.

**Figure 5.**
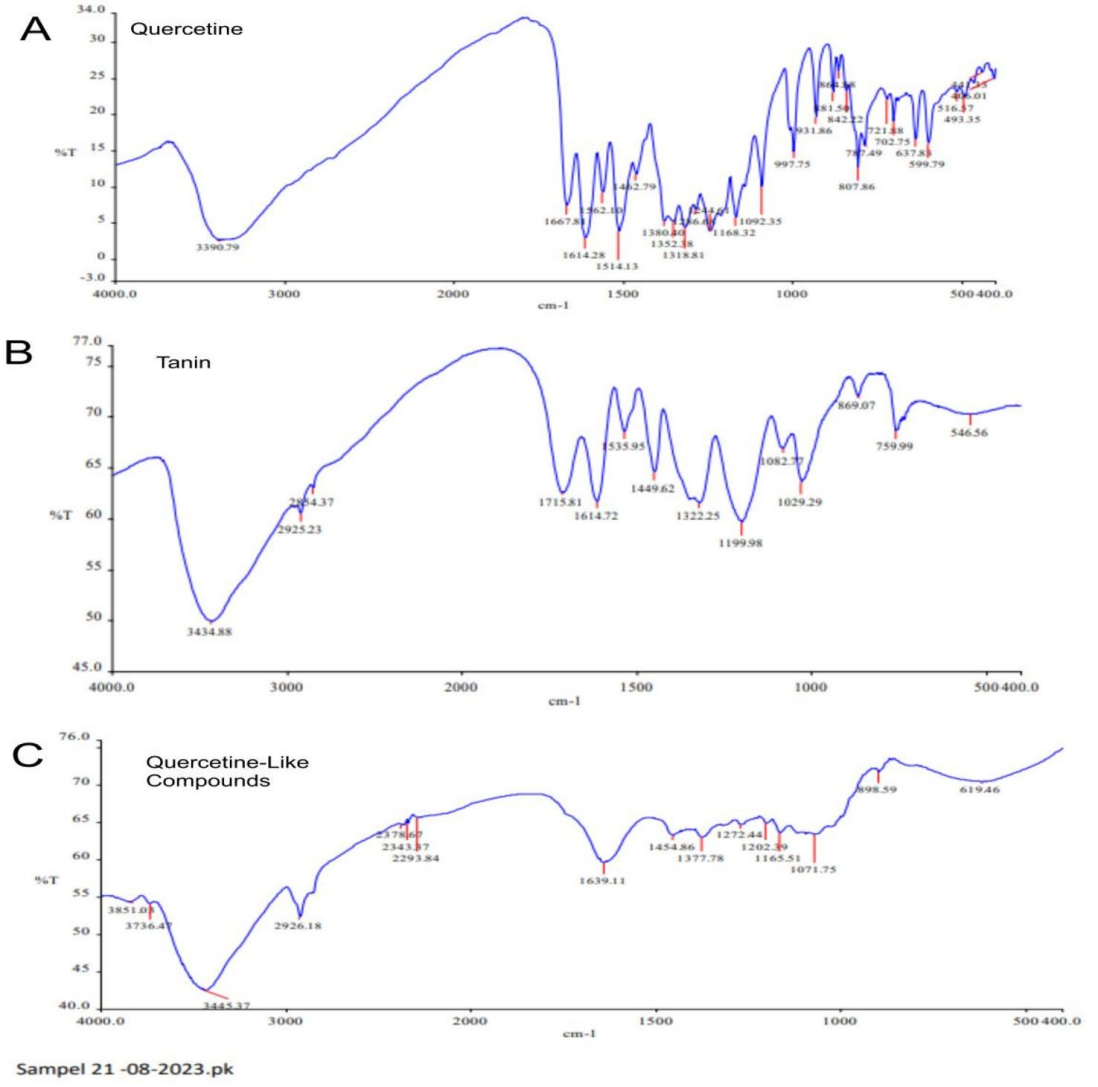
Fourier-Transform Infrared spectrophotometer wave numbers across a range of 4000 to 400 cm^−1^ with % transmittance (%T) with (A) quercetin standard; (B) tannin standard; and (C) quercetin-like compounds.

The results of the analyte molecular properties analysis compared to the quercetin standard using infrared (IR) electromagnetic radiation revealed that the wave numbers of the mid-IR spectral region (2.5–10 μm or 1500–600 cm^−1^) shared some similarities. Furthermore, an image of the FT-IR results is shown in Figure 5.

The spectrum of the QLCs compared to quercetin and tannin standards in the fingerprint area at wave values of 1600–400 cm^−1^ (V) is shown in Table 1. In general, in the near-infrared region, halogen compound elements with C–H stretching are often observed, as are C–O bonds or strong bending of C = C and alkenes. Whereas in the mid-infrared region, aromatic complexes, long double chains, and combinations with other ions are often observed as wagging, twisting, rocking, scissoring, and stretching.

**Table 1.** Spectrum infrared at the fingerprint area of quercetin-like compounds as the sample compared to the quercetin and tannin standards.

Spectrum infrared number	Wave number fingerprint area (V) at cm^−1^ and transmittance at %T						Classification of the infrared spectrum
	Quercetin-like compounds		Tannin standard		Quercetin standard		
1	619.46	70.45	None		637.88	16.61	Near-infrared
2	1071.75	63.41	1082.77	66.92	1092.35–997.75	10.10–14.86	Middle infrared
3	1165.51	63.64	1199.98–1082.77	59.70–66.92	1168.32	5.84	
4	1202.39	64.85	1199.98	59.70	1244.61–1168.32	3.94–5.84	
5	1272.44	64.76	None		None		
6	1377.78	63.02	None		1380.40	5.25	
7	1454.86	63.30	1449.62	64.58	1462.79	11.77	
8	1639.11	59.69	1614.72	61.71	1667.81–1614.28	3.02–7.54	

**Figure 6.**
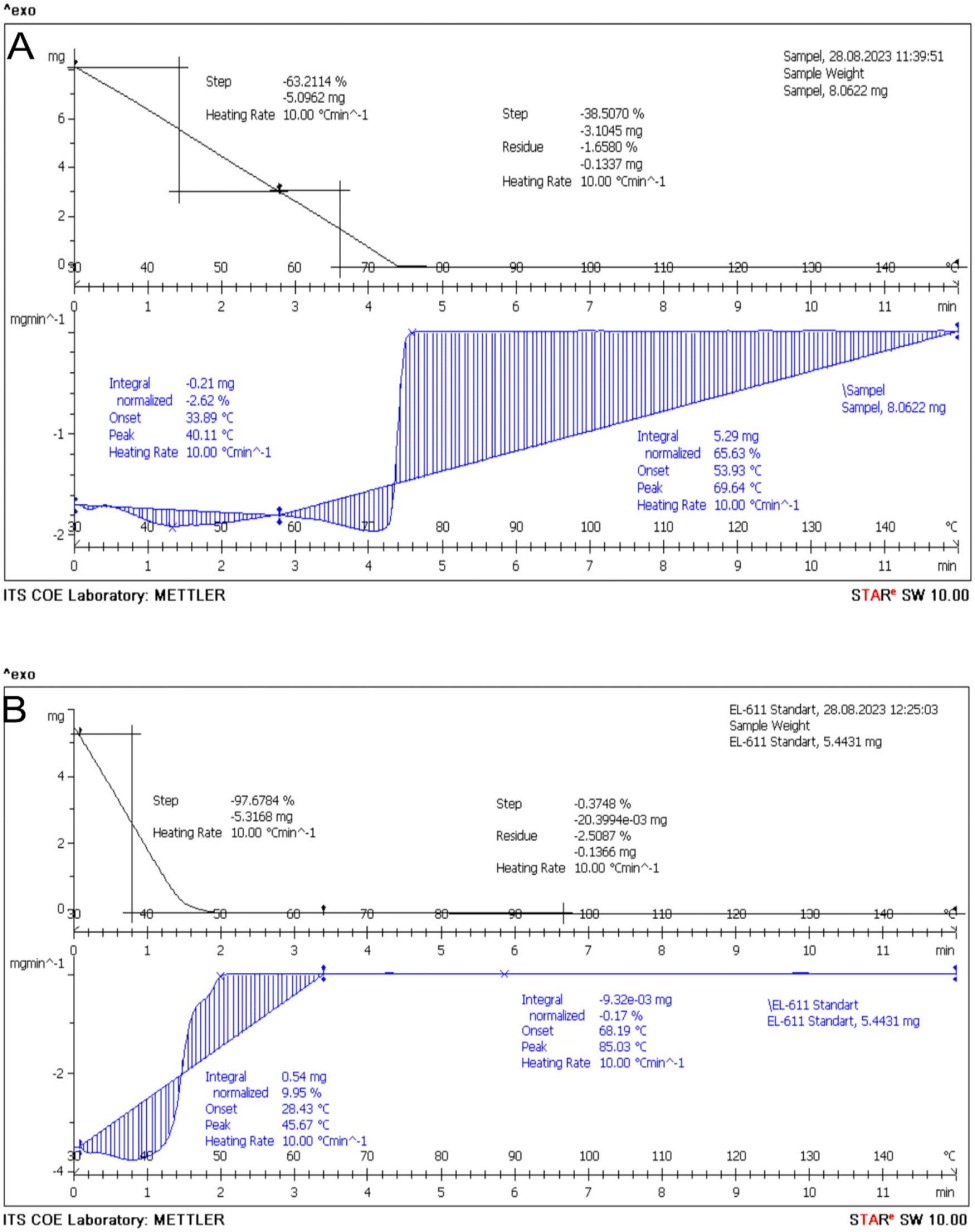
Thermal gravimetry-differential scanning calorimetry. Melting point of quercetin-like compounds (8.062 mg) (A) and the quercetin standard (5.443 mg) (B).

The TG-DSC analysis revealed that the start melting point of the QLCs was 4.2 min and a peak at 69.54 °C, which is much later compared to that of the quercetin standard. The melting point of the quercetin standard was available at 1.3 min, with a peak at 85.03 °C, as shown in Figure 6. The QLC sample was destroyed after 20 h. Unlike the standard, it begins to melt at 68.18 °C, comprising 0.17% of the total 5.443 mg and reaches a peak at 85.03 °C, or approximately 50 min.

**Figure 7.**
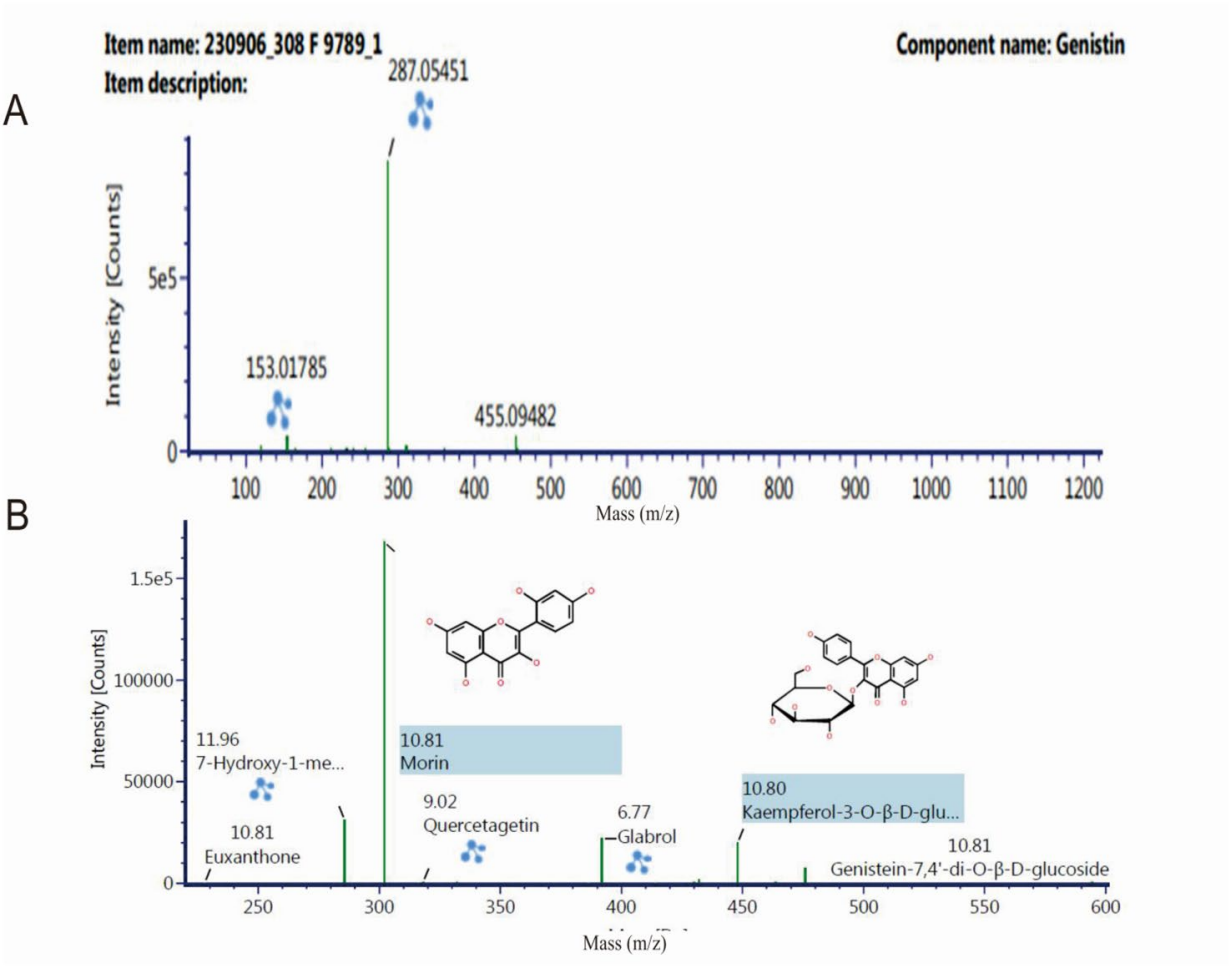
Spectrogram of genistein in methanol at 20 ppm at m/z 286.05 (A) and morin in methanol at 20 ppm at m/z 303.05 (B) using ultraperformance liquid chromatography-mass spectrometry with the UNIFI software program. The column was placed on octadecyl-xylene C_18_ stainless steel. System analysis used a gradient with a mobile phase of acetonitrile-water containing 0.1% formic acid.

Results from analysis using LC-ESI-MS showed that the analyte samples contained compounds, as shown in Table 2, with adducts at H^+^. The table shows the components with a fraction of more than one of the seven components, namely 5-hydroxy-6, 7-dimethoxyflavone-4′-O-β-D-glucoside, 7-hydroxy-1-methoxy-2-methoxy xanthone, euxanthone, geneistine-7.4′-di-O-β-D-glucoside, genistein, kaempferol-3-O-β-D-glucopyranoside, and morin. Efforts to analyze the similarities with the quercetin standard revealed 14 components, as shown in Table 2. LC-ESI-MS was used for screening, and no tannin content was observed. However, this does not indicate that the tannin element was lost, because the qualitative tannin content test still determined its presence before separation using a Preparative HPLC device. It is possible that when the analyte collector fraction was performed, the tannin compounds were not detected. It should be noted that the screening method using the LC-ESI-MS device in this study did identify tannin compounds with their fractional fractions, namely ellagic acid, and norbergenin. In this study, four components had a large response area (>1000 area chromatograms), namely, genistein, 7-hydroxy-1-methoxy-2-methoxy xanthone, kaempferol-3-O-β-D-glucopyranoside, and morin, with an average mass error of <2 for these four components. Two of the components are particularly interesting, namely genistein and morin. Genistein is often found in mistletoe plants. This compound has also been identified in experiments using equivalent working methods, including Preparative HPLC separation and isolating equipment with different specifications and columns, including different methanol-water mobile phases and eluent fractions (Medina et al. 2023). The separation and isolation device used in a previous study employed semipreparative HPLC with an analytical column. This rigid separation method uses a Preparative HPLC device with a column with special preparative specifications. The mobile phase fraction in the previous study was 70:30, and for the time it was performed using a 60:40 fraction (Lazuardi et al. 2023). The genistine and morin components were discovered to have a mass error rate of <2 with a large response area. Furthermore, the spectrograms of genistein and morin are shown in Figure 7.

**Table 2.** Fraction of quercetin-like compounds from the leaf extract of mistletoe of plant *Dendrophthoe pentandra (L.)* Miq. namely *Benalu duku.*

Component name	Formula	Observed of retention time (min)	Mass error	Total fragments fund	Isotope match mass-to-charge ratio (m/z) relative mass spectrum (ppm)	Isotope match intensity relative mass spectrum (%)	Response (area)
5-Hydroxy-6,7-dimethoxyflavone-4′-O-β-D-glucoside	C_23_H_24_O_11_	7.95	1.1	3	11.38	60.52	8471
7-Hydroxy-1-methoxy-2-methoxy xanthone	C_15_H_10_O_6_	11.96	−1.5	4	1.94	13.13	29,461
Euxanthone	C_13_H_8_O_4_	10.81	0.2	3	1.42	20.71	300
Geneistine-7.4′-di-O-β-D-glucoside	C_27_H_30_O_15_	10.81	−3.2	25	3.58	63.54	635
Genistein	C_21_H_20_O_10_	11.98	−2.2	6	2.13	15.12	1880
Kaempferol-3-glucoronide	C_21_H_20_O_12_	9.84	−2.1	1	2.58	29.16	239
Kaempferol-3-O-β-D-glucopyranoside	C_21_H_20_O_11_	10.80	−1.1	17	1.31	18.59	18,754
Morin	C_15_H_10_O_7_	10.81	−2.2	7	2.13	8.47	156,877
Ononin	C_22_H_22_O_9_	9.95	−6.1	1	6.24	36.8	380
Patuletin	C_16_H_12_O_8_	11.34	−1.6	1	2.07	33.49	620
Pedalitin	C_16_H_12_O_7_	12.14	−2.4	0	2.85	23.54	260
Pinnatifinosida A	C_21_H_18_O_9_	10.94	−6.4	0	6.57	33.25	325
Quercetagetin	C_15_H_10_O_8_	9.02	−1.5	0	1.94	33.15	706
Sinensetin	C_20_H_20_O_7_	16.70	−4.4	0	4.68	23.78	184

The results of the experiments revealed that the average random blood sugar value in the rats during the adaptation period of the first day was 106.667 mg/dL, while on the second day, it was 104.333 mg/dL, and on the third day of the adaptation period, it was 104.967 mg/dL. These results indicate that during the adaptation period of the rats, the random blood sugar value ranged between 104 and 107 mg/dL. The administration of streptozotocin at 0.045 mg/g body weight to rats induced the onset of diabetes and resulted in an increased average random blood sugar level of 535,233 mg/dL on the first day, and on the second day of administration, the average random blood sugar level was 540,767 mg/dL. Postoral administration of the analytes at 0.5 mg/kg body weight twice daily for up to 5 consecutive days produced the following average random blood sugar values for 14 days postadministration of the analytes: 492.266 mg/dL; 476.866 mg/dL; 463.370 mg/dL; 448.900 mg/dL; 430.066 mg/dL; 404.833 mg/dL; 388.833 mg/dL; 374.5 mg/dL; 360.366 mg/dL; 342.8 mg/dL; 323.2 mg/dL; 309,433 mg/dL; 290,966 mg/dL; and 273,833 mg/dL. Furthermore, the results of the analyte tests on diabetic rats are presented in Table 3. The random blood sugar values in rats after analyte treatment are expected to continue to decline; however, in this study, evaluations were only performed until day 14. A decrease in random blood sugar levels in the rats was followed by a recovery of body condition in the form of increased agility and appetite. The assessments did not cause death in any of the rats during the experiment. However, death might occur during the analyte testing period if the oral administration *via* the oral sonde is performed incorrectly. In such circumstances, analyte fluid can enter the lungs. The results of the statistical analysis using the different paired *t*-tests of the before–after method showed that the average random blood sugar values before the oral administration of the analytes was 538 mg/dL, and the average random blood sugar values decreased after the administration of the analytes to 384.27 mg/dL (*p* < .05). The test also showed that the mean of the paired samples tested was 153.731 mg/dL, with a standard deviation of 42.566 mg/dL and a standard error of the mean of 7.772 mg/dL. The confidence interval was 95% with a lower level of 137.836 mg/dL and an upper level of 169.625 mg/dL, with *t*** **=** **19.781, and 29 degrees of freedom.

**Table 3.** Experimental quercetin-like compounds (QLCs) were used for experiments on diabetic rats.

Before the administration of QLCs (days)			After the administration of QLCs (days)															Analysis statistics
1st	2nd	Mean ± % CV^a^	1st	2nd	3rd	4th	5th	6th	7th	8th	9th	10th	11th	12th	13th	14th	Mean ±% CV^b^	*p*
522	524	523 ± 0.27	450	425	431	400	388	387	386	386	355	350	345	321	316	302	423.07 ± 11.96	<.05
540	550	545 ± 1.29	528	488	486	430	425	423	419	417	415	412	389	385	355	351	447 ± 11.71	
511	520	515.5 ± 1.23	499	485	481	479	476	475	444	441	432	424	421	411	402	388	370.14 ± 7.90	
540	542	541 ± 0.26	471	430	441	420	411	386	366	362	331	317	318	316	312	301	318 ± 15.25	
530	533	531.5 ± 0.40	420	419	417	413	410	302	301	289	287	267	250	249	220	208	309 ± 25.27	
533	531	532 ± 0.26	410	408	400	385	375	320	305	288	285	264	240	230	215	201	353.79 ± 24.27	
560	575	567.5 ± 1.87	510	499	440	390	385	381	375	317	315	290	288	268	255	240	344.64 ± 25.51	
573	575	574 ± 0.24	501	458	420	378	365	355	340	320	310	305	285	275	260	253	362.93 ± 21.59	
540	550	545 ± 1.29	489	485	477	465	398	388	370	360	320	310	299	260	245	215	418.50 ± 25.49	
512	518	515 ± 0.82	509	489	477	468	455	437	420	415	410	405	388	356	320	310	377.50 ± 14.41	
560	563	561.5 ± 0.37	498	478	465	455	430	410	380	375	355	320	305	289	270	255	375.64 ± 21.49	
578	574	576 ± 0.49	501	488	455	432	428	399	387	386	355	320	310	287	256	255	367.29 ± 21.64	
540	545	542.5 ± 0.65	510	505	487	486	433	386	377	364	352	289	250	249	233	221	355.29 ± 28.86	
570	579	574.5 ± 1.11	467	453	450	430	427	388	357	314	309	300	280	277	267	255	403.79 ± 21.99	
518	520	519 ± 0.27	511	489	477	465	455	431	422	398	387	375	360	355	278	250	401.86 ± 18.99	
522	524	523 ± 0.27	501	497	488	485	475	423	399	396	393	384	320	301	299	287	360.57 ± 19.03	
530	532	531 ± 0.27	497	484	421	411	396	377	363	354	320	311	305	288	266	255	391.43 ± 20.95	
535	536	535.5 ± 0.13	501	499	480	460	420	410	392	385	377	374	318	299	286	279	410.29 ± 19.47	
549	550	549.5 ± 0.13	500	489	477	467	455	448	420	399	387	367	355	340	330	310	385.43 ± 15.48	
568	570	569 ± 0.25	499	487	486	477	465	421	397	365	344	320	318	299	268	250	409.57 ± 22.76	
566	567	566.5 ± 0.12	478	469	468	466	445	420	415	401	397	387	377	369	355	287	383.93 ± 13.14	
500	510	505 ± 1.40	499	477	465	453	441	410	398	387	359	340	311	302	288	245	423.86 ± 20.49	
536	539	537.5 ± 0.39	511	505	499	487	468	455	439	421	404	388	367	358	333	299	405.93 ± 16.04	
521	522	521.5 ± 0.13	507	498	477	466	450	420	397	385	371	366	351	341	333	321	419.21 ± 15.67	
513	534	523.5 ± 2.84	551	541	522	503	496	477	460	420	410	316	302	299	295	277	381.36 ± 24.33	
552	553	552.5 ± 0.13	503	495	485	475	431	406	377	365	344	321	307	288	276	266	397.21 ± 22.33	
516	521	518.5 ± 0.68	502	488	477	472	431	411	399	388	377	365	344	321	299	287	393.21 ± 17.73	
513	514	513.5 ± 0.14	449	442	430	422	413	401	395	394	387	382	375	365	335	315	392.50 ± 9.70	
501	521	511.0 ± 2.77	499	491	487	486	450	401	388	377	368	364	321	299	287	277	370.71 ± 20.25	
508	531	519.5 ± 3.13	497	445	443	441	405	397	377	366	355	351	297	286	275	255	423.07 ± 19.70	

*Note:* The column of the mean before the administration of QLCs with superscript a versus the mean after the administration of QLCs with superscript b were significant at *p* < .05.

## Discussion

Analysis of the IR spectrum of the analytes revealed fingerprint areas of 600 and 1500 cm^−1^, as shown in Figure 5 and Table 1. This demonstrates that V is 619.46 and 637.88 cm^−1^ of analytes and quercetin standard near the IR region and were alkynes or –C≡C–H: C–H bends with a broad and sharp spectrum, respectively, and exhibited no partial tannin compounds. Finding no partial tannin components can result from a long isolation process, which eventually removes part of the tannin. Spectrum at V 1071.75, 1082.77, 1092.35, and 997.75 cm^−1^ of the analytes, tannin standard, and quercetin standard were C–C, C–O, and C–N results. In this middle IR region (1071.75, 1082.77, 1092.35, and 997.75 cm^−1^) analytes were not specifically determined, probably because of the presence of long chains, single aromatic compounds, or complex aromatic molecules (Kokalj Ladan et al. 2017; Başaran et al. 2022). Bands in the region of 1250–1000 cm^−1^ were because of C–H in-plane bending at the C within the spectrum of a single aromatic, although these bands are often too weak to be observed in most aromatic complex compounds. The V of 1165.51, 1199.98–1082.77, and 1168.32 cm^−1^ of the QLCs, tannin standard, and quercetin standard have similarities with other substances as an aromatic compound (Hayat et al. 2020). The V of 1202.39, 1199.98, and 1244.61–1168.32 cm^−1^ for the QLCs, tannin standard, and quercetin standard were related to the C–O stretch spectrum. Figure 5(A–C) is the R–OH in the middle IR area. The IR spectrum of the analytes at 1272.44 cm^−1^ does not appear to match that of the tannin and quercetin standards and is likely to be an overtone and combination band in-ring compounds. However, the V of 1377.78 cm^-–1^ in the analytes and 1380.40 cm^−1^ in the quercetin standard were identical to the O–H bend at the sharp spectrum level as a carboxylic acid compound. The spectra of 1454.86 cm^−1^ in the analytes, 1449.62 cm^−1^ in the tannin standard, and 1462.79 cm^−1^ in the quercetin standard have identical C–O stretching performance as carboxylic acid compounds. The carbonyl stretch of esters appears at higher wave numbers than the carbonyl stretch of normal ketones or bonds of the aromatic complex compounds, such as naphthalene and anthracene. In aliphatic esters, this stretch is in the range of 1639.11–1614.28 cm^−1^ (spectrum-specific band of acetate compounds, Figure 5(A–C)). In other cases, for the α and β functional groups, such as ion nitrate and amine, or combined with esters, ketone, and in-ring compounds, the stretch was discernable from 1730 to 1715 cm^−1^. Thus, in Figure 5(A), there will be more than one compound that has a molecular core structure similar to quercetin or a functional group similar to the bond in the quercetin standard (Lazuardi et al. 2022).

The TG-DSC analysis of the analytes in Figure 6(A) reveals that the initial sample weight was 8062 mg and was reduced to 4681 mg (approximately 41.93%) over 264 s. This was different when compared with the standard, as shown in Figure 6(B), where 86 s was required to reduce the weight of the initial quercetin standard (5.4431 mg) to approximately 2.282 mg (41.93%). This indicates that there are other compounds in the analytes, and these other compounds have the above melting properties. Given that the main components of the two compounds originate from plants dissolved in polar solvents, the two compounds, analytes, and quercetin standard, quickly melted in a shorter amount of time, as shown in Figure 6(A,B). The quercetin standard (Figure 6(B)) melts faster than the analytes because of its fairly high purity. The results of the TG-DSC analysis also guarantee that the results of the isolated analyte do not require excessively high temperatures at the melting point limit (approximately 75 °C). Thus, the analytes obtained are not damaged at high temperatures (Shaltout et al. 2011; Boguta et al. 2017).

The results of the molecular ion mass for the analytes revealed that the analytes consist of more than one compound, as presented in Table 3. Table 3 shows a large response for morin, 7-hydroxy-1-methoxy-2-methoxy xanthone, kaempferol-3-O-β-D-glucopyranoside, and genistein. Fourteen components in one unit, referred to as analytes, were predicted when performing FT-IR and TG-DSC analyses of the analytes (Huang et al. 2021; Tsioptsias and Tsivintzelis 2022). The components that form analytes can be in the form of long-chain or aromatic structures, or a combination of both. However, in principle, all analytes have the requirements to be monitored by infrared detectors and mass molecules, including the physical characteristics of TG-DSC (Trivedi et al. 2020). The genistein compound is a flavonol compound that has been found in BD extracts as a result of previous research and has antiviral properties (Saha and Kroon 2020; Başaran et al. 2022). Previous research reports used different maceration separation techniques, including isolation using methanol solvent, followed by ethyl acetate solvent, and n-hexane solvent. In this study, methanol was used as the solvent, followed by a mixture of water and n-hexane. The difference in the liquid–liquid extraction technique with the use of ethyl acetate is that it reduces the content of analytes other than genistein. Morin is a new compound discovered in this study. Morin and genistein are thought to strengthen each other in maintaining the survival of eukaryotic cells. The elements of these two compounds that have the potential to sustain eukaryotic cells are organic ion elements (C, H, O, N, S, P, and K) (Garbiec et al. 2022; Gor et al. 2022). The organic ion element in LC-ESI-MS monitoring is found in component 14, as shown in Table 3. Genistein is known to have relatively stable bonds and does not break easily in molecular-based monitoring compared to morin. Therefore, in Figure 7(A), it appears that genistein is single. This is different from morin or the components 7-hydroxy-1-methoxy-2-methoxy xanthone and kaempferol-3-O-β-D-glucopyranoside (Periferakis et al. 2022). Other components, as shown in Figure 7(B), tend to have large fractions, thus indicating unstable bonds between the ions. In principle, molecules with unstable structures have weaker pharmacodynamic capabilities. These limitations can be added to other external elements that ultimately produce semisynthetic secondary metabolites. The glabrol compound shown in Figure 7(B) has a retention time of 6.77 min but is not included in Table 3, revealing that it has an area response that cannot be quantified by the LC-ESI-MS device despite being recognized by the device. Thus, it was not included in the summary list for the LC-ESI-MS device.

The final experiment included diabetic rats, in which the average random blood sugar levels (mg/dL) were monitored daily. Before treatment, the maximum percent coefficient variation was 9.00 for random blood sugar levels. The random blood sugar values before treatment showed a percent coefficient of variation of <10. Thus, the random blood sugar values of the rat under normal conditions are within this range. The random blood sugar values of the diabetic rats on day 2 after administration of streptozotocin were five times greater than those in normal rats. Five days after the oral administration of analytes twice a day, the random blood sugar values in the first five days did not noticeably decrease. However, 14 days after administration, a marked decrease was observed. The decline continued at a constant rate, which was >1 mg/dL of random blood sugar levels every day. At the 14-day observation, the random blood sugar was reduced (Alam et al. 2022). The correlation analysis between random blood glucose values from the before–after analysis in the same rats showed that the correlation was not especially strong. However, if the observation period was extended to one month, it is estimated that the correlation value from before and after the analyte administration would produce a significant linkage. However, observation over the 14 days, followed by analysis using a paired *t*-test using the same rats, produces a real difference in Table 3. The results of the study showed that the analyte administration tended to reduce the random blood sugar levels, which was influenced by the internal compounds of oral therapeutic agents as analytes after interacting with physiological compounds in the rat’s body that regulate the body’s blood sugar levels. Polyphenol elements that have the potential to lower blood sugar levels are (1) genistein with its derivatives, (2) 5-hydroxy-6,7-dimethoxyflavone-4′-O-β-D-glucoside, (3) morin, (4) quercetagetin, (5) and tannin. These five compounds were selected considering that (a) they have the highest levels in the analytes and (b) genistein often appears in analyte preparations. The five elements are strongly electronegative, and they can protect the cytoplasm of pancreatic β-cells. When the five elements are bound to the ion part of the induction group of pancreatic β-cells, the internal balance in the cell occurs, thereby ensuring that the cell does not rupture easily. The strong bond has a strong potential energy, and it is not easy to undergo a rearrangement reaction, which results from addition or substitution reactions. The additional modifications of the other elements will generally be created using The Quantification Structure-Activity Relationship (Q-SAR) theory. This technique eventually produces new drug molecules in the form of semi-synthetics. This semisynthetic manufacturing technique reduces the utilization of secondary metabolites from natural plants. In principle, semisynthetic compounds are 50% similar to medicinal plants, and fifty percent are an inexhaustible chemical synthesis of these materials. If the semisynthetic model can be developed, additional new molecular derivatives will be obtained, and the availability of new drugs will be manufactured and put on the market faster. In addition, the incidence of unfavorable risks because of drug use is decreasing. The limitation of this study is that the experiment was only performed on the 14th day after the oral administration of analytes. Thus, the observational results have not been able to establish the reduction of the random blood sugar levels to normal conditions. However, the tendency for the random blood sugar levels to decrease over 14 days of observation has been demonstrated.

## Conclusion and recommendations

A general analysis of the QLCs analytes in diabetic rats showed that an oral dose of 0.5 mg/kg body weight twice a day for five days can reduce the random blood sugar levels (*p* < .05). The analyte compounds in question are produced from the stratified maceration of BD leaves growing in *Lancium domesticum*. The recommendation for this study was that, for the application of analytes for oral preparations, the acid–base balance must be maintained and must be free from germs and fungi, which may appear in natural preparations, such as analytes from BD leaf extract.

## Data Availability

Underlying data Figshare: Lazuardi et al. (2023). Quercetin-like compounds of mistletoe extract as an anti-diabetic substance in rats suffering from diabetes. figshare. Journal contribution. https://doi.org/10.6084/m9.figshare.24546796.v6. This project contains the following underlying data: Proof Figshare (The first submitted with grammar and substantial correction from ENAGO Corp.)
Figur-1 600.jpg Data FT-IR.PDF S.308.R.59 NP Screening QToF ESI - All identifications Kromatogram pdf 2.96 MB adc31dba4579b9401946233edd7a9ae4 S.308.R.59 NP Screening QToF ESI + All identifications Kromatogram pdf 3.46 MB 88a99e0495d21530f77dddad1d47e4f0 S.308.R.60 NP Screening QToF ESI - All identifications Kromatogram pdf 828.86 kB 46d9f6a3b25ff053471774b0e91a7b02 S.308.R.60 NP Screening QToF ESI + All identifications Kromatogram pdf 2 MB 469fae57098ecd4e69009b5c75be7d6a S.308.R.61 NP Screening QToF ESI - All identifications Kromatogram pdf 959 kB 6ed48d4f826f0f2c9e08dfce486c1d27 S.3008.R.61 NP Screening QToF ESI + All identifications Kromatogram pdf 1.27 MB 8b866fe25fed2372d8dd6151d45d7972 IKA LEL-ITS TGA pdf 3.24 MB 128013edfaa071a18d7678f9fb831628 Ekstrak Benalu Duku + STD quercetin 10 PPM pdf 403.38 kB ff6aefd06dec4b15da393fc89a461097 spesifikasi LC Prep 1260 Infinity II with DAD pdf 1.12 MB ac90bb32ee799bd8b5f804b5f31b0cbd Before-After spv 6.4 kB 3fe25970256b733e361e31ad5670fe46 Excel Trials to rats xlsx 10.08 kB 2dc199a21418674f4d53d1be4708f567 OneTouch-Ultra2-User-Guide pdf 1.23 MB 311ca471e1649c699a663eb5a227cdd4 Trials-edit1 xlsx 12.88 kB a021d576376b3e52ece2de8ac97d7168 Animal Ethic Clearance pdf 816.14 kB 4cc7e2977cf940c6d9ef0f761e966d40 Data Mistletoe LIPI pdf 187.19 kB 1e77d9e129b2ab3231904f7dcab6f1cf LUTVIANA RACHMAWATI - 061911133046 (2) pdf 1.03 MB 325e7eaea373f037ba09372054dddfec Response_Reviewer_I pdf 180.5 kB 810a47c291607a312aae760b559670c7 Manuscript_Edit_5_April_2024 docx 1.29 MB c37fd4ad91e1786ee84893e025238056.
